# Design and Validation of a Multiplex KIR and HLA Class I Genotyping Method Using Next Generation Sequencing

**DOI:** 10.3389/fimmu.2018.02991

**Published:** 2018-12-19

**Authors:** Laia Closa, Francisco Vidal, Maria J. Herrero, Jose L. Caro

**Affiliations:** ^1^Histocompatibility and Immunogenetics Laboratory, Blood and Tissue Bank, Barcelona, Spain; ^2^Transfusional Medicine Group, Vall d'Hebron Research Institute, Barcelona, Spain; ^3^Congenital Coagulopathy Laboratory, Blood and Tissue Bank, Barcelona, Spain; ^4^CIBER of Cardiovascular Diseases, Madrid, Spain

**Keywords:** KIR, HLA, next generation sequencing, genotyping, NGS analysis

## Abstract

Killer cell immunoglobulin-like receptors (KIR), considered the most polymorphic natural killer (NK) cell regulators, bind HLA class-I molecules or still unknown ligands. Interest in KIR genotyping is increasing because of the importance of these receptors for identifying the best possible donor in hematopoietic stem cell transplantation to obtain a graft-versus-leukemia effect. Currently, routine protocols to determine the gene content of the KIR cluster are exclusively performed by PCR-SSO and PCR-SSP. To improve the study of these genes, we developed a multiplex, long-range PCR strategy suitable for simultaneous, high-resolution HLA class I and KIR genotyping by next generation sequencing (NGS). This protocol allows amplification of the 14 KIR genes, 2 KIR pseudogenes, and HLA class I genes, with subsequent sequencing on an Illumina platform. The bioinformatics analysis for KIR genotyping was performed by virtual hybridization of gene-specific probes, and HLA genotyping was done by GenDx NGSengine software. To validate the method reliability, 192 genomic DNA samples previously characterized by PCR-SSO were used. When a specific KIR gene was present, a large number of gene-specific virtual probes were detected, whereas when it was absent, very few or none were found, enabling cutoff establishment. Concordance for both the KIR and HLA assignments as compared with the previous characterization was 100%. In conclusion, the multiplex PCR NGS-based strategy presented could provide an efficient, less costly method for KIR-ligand genotyping by gene presence/absence. Furthermore, allele resolution will be possible when KIR-specific software becomes available.

## Introduction

Natural killer (NK) cells are lymphocytes with a critical role in innate immunity by protecting against viral infection and malignant cells (1). This function seems to be controlled by a complex network of surface receptors and the interactions with their ligands. Among these receptors, killer cell immunoglobulin-like receptors (KIR), which belong to a family of type I transmembrane glycoproteins expressed on NK cells, are considered the most polymorphic and one of the most important regulators of these cells (2). KIR proteins can tune potent effector functions by mediating human NK cell cytotoxicity via activating or inhibiting signals resulting from selective binding with human leukocyte antigen (HLA) class-I molecules and some still unknown ligands (3, 4). This group of receptors prevents NK cell-mediated attack against normal autologous cells, whereas cells in which HLA class I expression is compromised by tumor transformation or viral infection become susceptible to NK-mediated killing. The anti-leukemia effect of donor NK cell alloreactivity in KIR ligand-mismatched haploidentical (partially-matched related donor) hematopoietic stem cell transplantation (HSCT) may provide curative therapy for many patients. However, the impact of KIR allele polymorphism on NK cell alloreactivity remains to be investigated (5). The KIR locus, which contains a family of polymorphic and highly homologous genes, is located on chromosome 19q13.4 within the leukocyte receptor complex. In contrast to HLA class I genes, that are defined purely on allelic polymorphisms, the KIR gene complex is both polygenic and extremely polymorphic. The structural variation creates multiple gene-content haplotypes. In addition, extensive allelic variability has been identified, particularly in the inhibitory genes (6). Variability in gene-content haplotypes is responsible for significant diversity both within and between populations (7). This greatly differences between individuals are explained because KIR genes have evolved rapidly by mutation and recombination to generate multiple alleles at every locus and the KIR-gene content appear at different and varying frequencies in each population (8).

**Figure 1:**
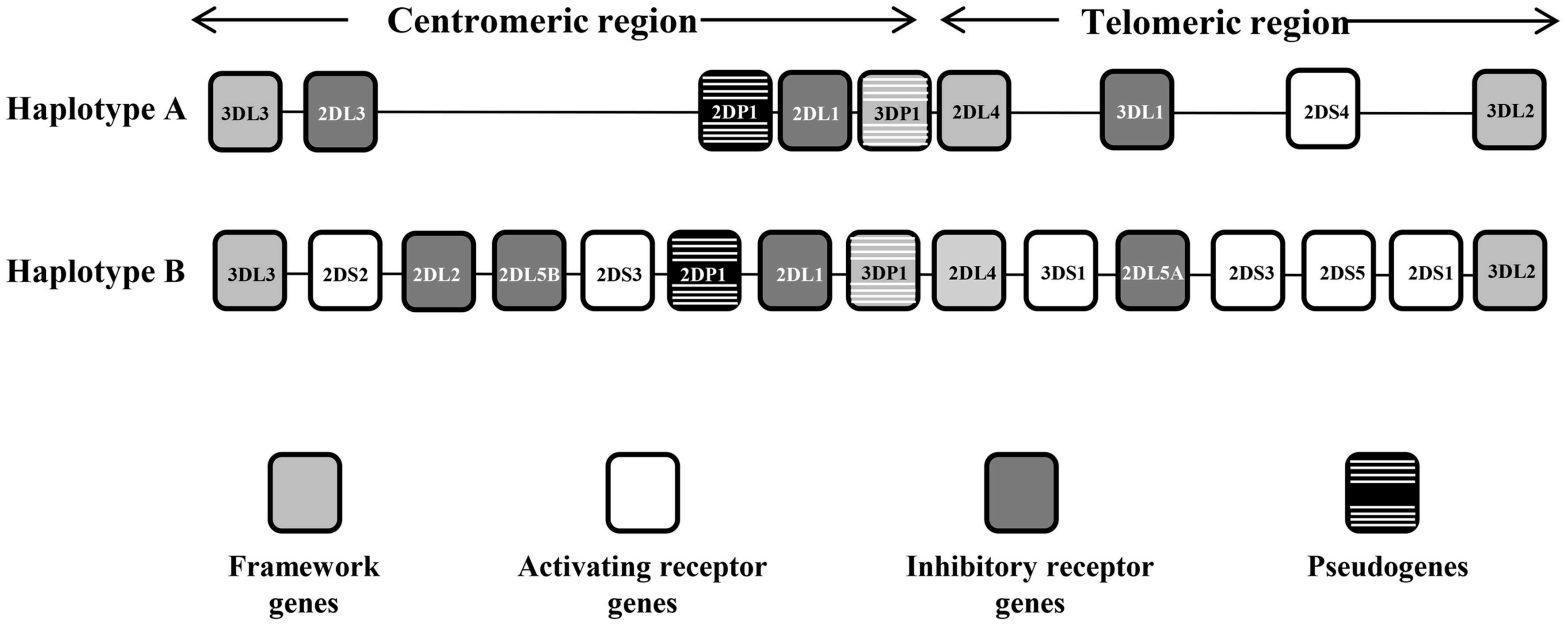
Representative KIR A and B haplotypes. The KIR gene order is schematized for the A and B haplotypes. The framework genes delimiting the centromeric and the telomeric regions are indicated by gray boxes, the activating genes by white boxes, the inhibitor genes by dark gray boxes, and the pseudogenes by black boxes with white stripes. There are some instances where a pair of KIR gene names was combined to form one name after it became apparent that they occupied the same locus—these are KIR2DL2/3, KIR3DL1/S1, and KIR2DS3/5, which act as alleles of the same locus. KIR2DL5 as well as KIR2DS3/S5 can be located on either/both the centromeric region or the telomeric region of the KIR complex.

The KIR system is composed of 13 genes and 2 pseudogenes, four of these genes are constitutive and are always present in the genome of all individuals, and 10 are variable, (i.e., they may be present or not in the genome). These genes are 9 to 16 Kb in size and contain 8 or 9 exons that encode the signal peptide, 2 or 3 extracellular domains, a stem region, a transmembrane region, and a cytoplasmic tail (9). KIR gene content has been organized into 2 haplotypes, A and B. Haplotype A is invariant in terms of gene content and has only 1 activating KIR gene (*KIR2DS4*), whereas haplotype B is quite variable and has additional activating genes (Figure 1). Moreover, the distribution of receptors in the NK cell is stochastic; each NK clone may express 1 or more receptors and show increasing transcriptional complexity. Finally, the largest contributor to the diversity of the KIR region is its elevated gene polymorphism, with 907 currently defined alleles (Release 2.7.0 July 2017) (10, 11).

KIR genotyping is usually performed on the request of a specialized physician for fertility studies or for haploidentical hematopoietic stem cell transplantation. The methods currently used to determine the KIR gene content are PCR-SSO, based on amplification of the locus of interest by PCR followed by hybridization with gene-specific oligonucleotide probes, or PCR-SSP, consisting in gene-specific PCRs to determine the presence of each gene (12, 13). These strategies provide information in low resolution, genotyping the KIR gene system by determining the presence or absence of genes. Conversely, NGS technology has great potential for detecting allelic variants, as has been demonstrated in HLA typing, in which a large number of samples can be efficiently analyzed simultaneously (14).

In this study, we aimed to develop and validate an economically viable method to genotype receptors and ligands simultaneously by taking advantage of the shared regions of most KIR genes, and obtain information about all the KIR loci and the classic HLA class I genes.

## Material and Methods

### Samples

In total, 192 samples were selected: 30 samples of genomic DNA from the 10th International Histocompatibility Workshop (IHW), known for their high-resolution KIR and HLA class I and II genotyping, and 162 genomic DNA samples from the BST Histocompatibility and Immunogenetic laboratory, well-characterized by PCR-SSO for KIR content and by PCR-NGS and/or SBT for high-resolution HLA class I genotyping. All individuals gave written informed consent, in accordance with the protocols approved by the Barcelona Blood and Tissue Bank ethics committee.

### Method Validation

Four runs were performed to validate the method. The first 2 runs (96 samples/run) were sequenced using the 300-cycle MiSeq reagent kit, and the other 2 runs (78 samples/run) were performed with the 500-cycle MiSeq kit to enable comparison of the results obtained with different read lengths.

### KIR and HLA Class I Multiplex Long-Range (LR) PCR

The QIAsymphony DNA kit was used for genomic DNA extraction. DNA concentration in all samples was measured using a NanoDrop Lite spectrophotometer (Thermo Scientific) at 260 nm.

**Figure 2:**
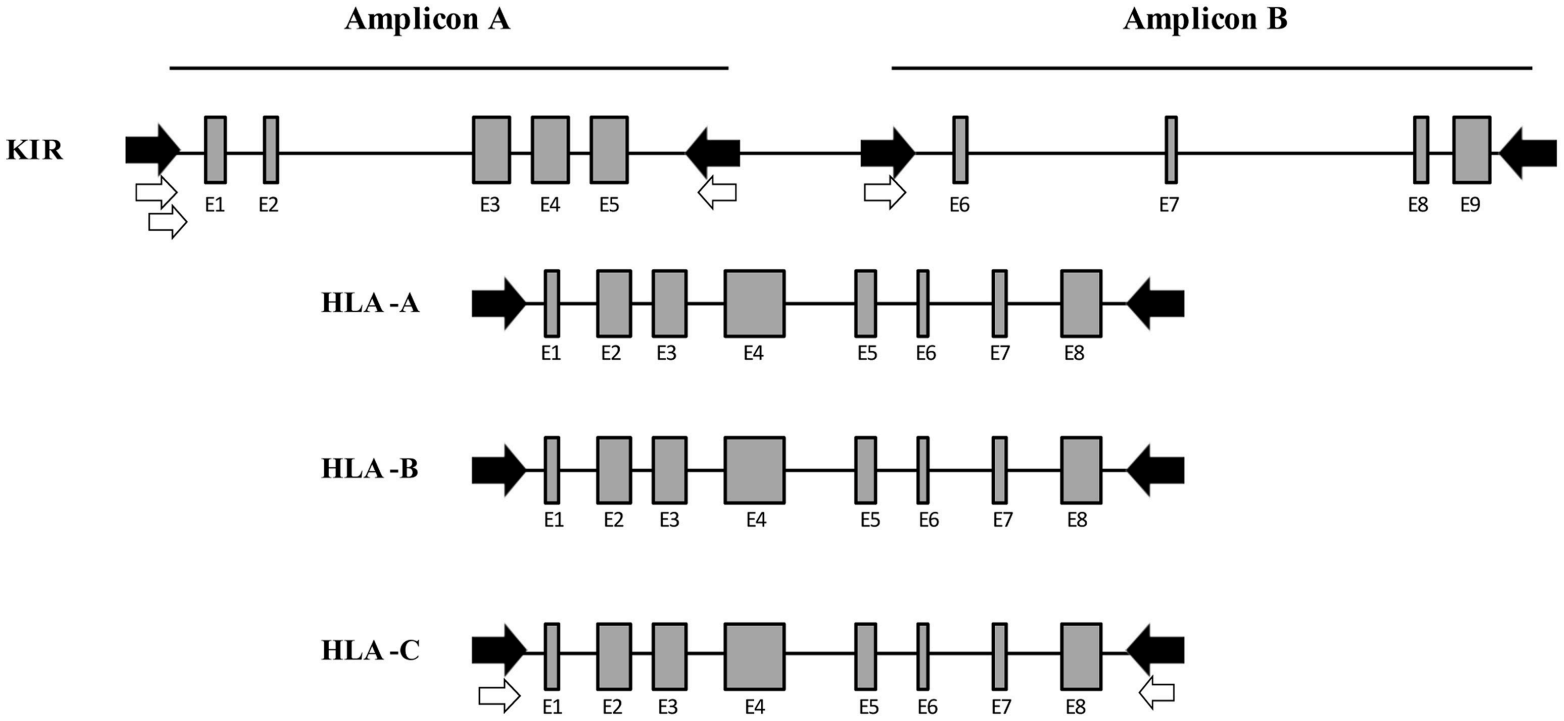
Multiplex long-range PCR for KIR and HLA class I gene amplification. Four generic in-house-designed primers (in black) were used to obtain 2 amplicons whose length was similar to the HLA class I amplification, covering the entire KIR locus with the exception of intron 5. Four KIR gene specific primers (in white) were designed after generic amplification failed due to a few mismatches in the primer binding sites. For Amplicon A, 2 specific forward primers were designed for *KIR2DL4* and *KIR3DL3*, respectively. Downstream of exon 5, 1 specific reverse primer was designed for *KIR3DP1*. For Amplicon B, 1 specific primer was designed for *KIR3DL3*. Moreover, 4 in-house-designed pairs of primers were used to amplify the entire HLA class I (*HLA-A, -B, and -C*).

To amplify the total of KIR genes and pseudogenes, 8 primers were designed to obtain 2 amplicons having a length similar to that of the class I HLA genes and covering around 80% of the entire KIR cluster. Multiple alignments of KIR reference sequences from the Immuno Polymorphism Database (IPD; see Web Resources) were used to delimit the best regions to specifically amplify all genes comprising the KIR system simultaneously. For amplicon A, which covers exons 1 to 5, 3 forward primers were designed, 1 generic primer for all KIR genes and 2 specific primers for *KIR2DL4* and *KIR3DL3*, respectively. Downstream of exon 5, 2 reverse primers were designed, 1 generic for all KIR and 1 specific for *KIR3DP1*. For amplicon B, which covers exons 6 to 9, 2 forward primers were designed, 1 generic and 1 specific for *KIR3DL3*. Finally, 1 reverse primer was designed downstream of exon 9 to generically target all KIR genes. Using this method, only intron 5 remained partially uncovered. The expected amplicon length varied according to each KIR gene, and ranged from 3,800 to 8,000 bp. This wide range is due to the considerable structural diversity of the KIR alleles, which include various duplications and deletions. These 8 KIR primers plus 4 previously designed in-house primer pairs to amplify the full length of *HLA-A, HLA-B*, and *HLA-C* comprised a mix with a total of 16 primers for simultaneous amplification of 19 loci (Figure 2).

**Figure 3:**
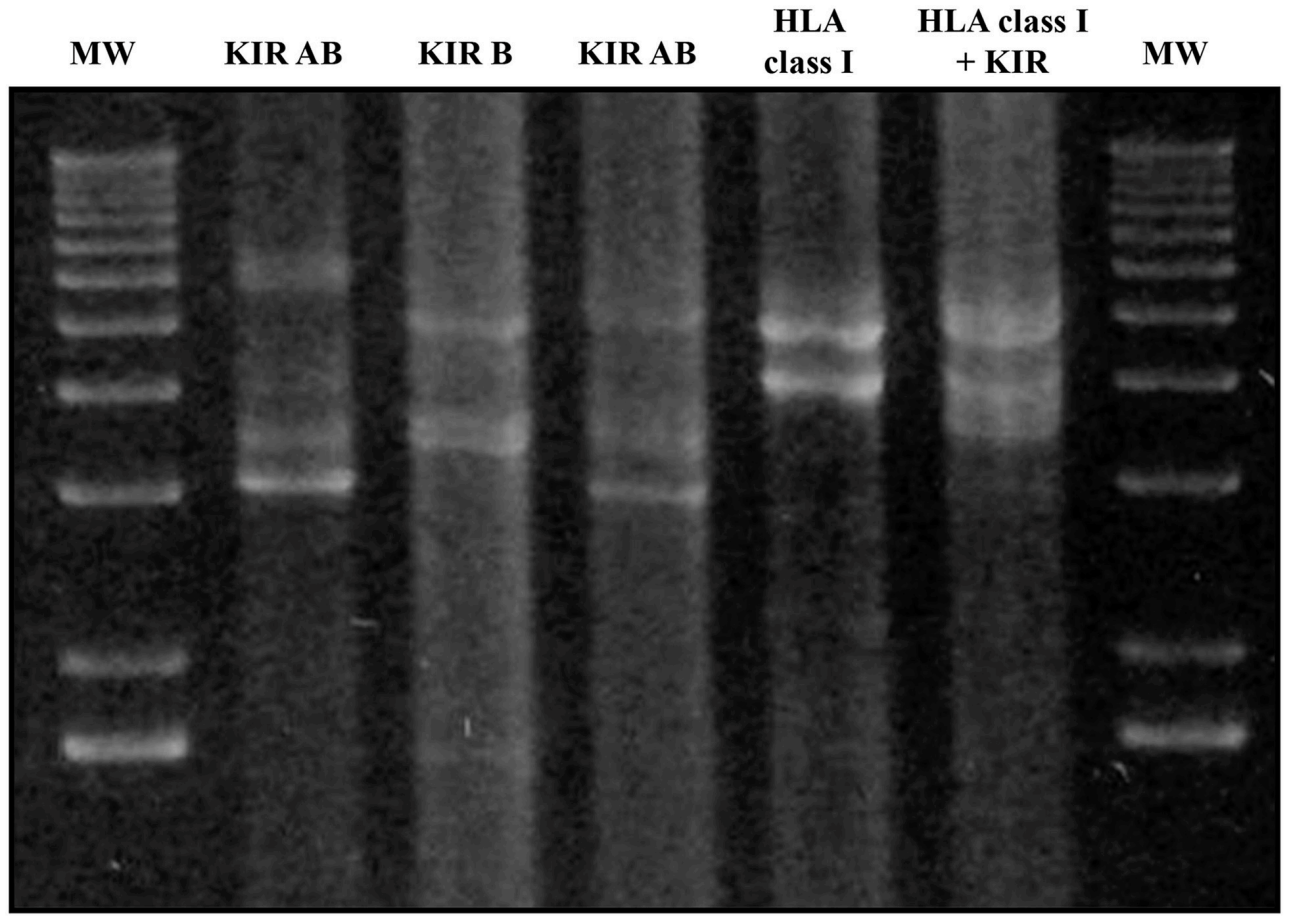
Agarose gel electrophoresis (1%) of KIR and HLA class I multiplex long-range amplifications. Lines 1 and 7: molecular weight (MW); Line 2: Amplification of all KIR gene Amplicon A; Line 3: all KIR gene Amplicon B; Line 4: Amplicons A + B of all KIR genes; Line 5: HLA class I amplicon; Line 6: Both KIR amplicons and HLA class I. Amplicon A lengths varied from 3,000 bp in the case of *KIR2DL4* to 7,000 bp. Amplicon B varied from 3,600 to 5,400 bp in the case of *KIR3DL3*. Amplicon lengths for *HLA-A, HLA-B*, and *HLA-C* were 4,900, 4,000, and 4,100 bp, respectively.

Multiplex LR-PCR amplification was established and optimized for best performance using the SequalPrep Long PCR Kit with dNTPs (Invitrogen, Carlsbad, CA, USA). Amplification was carried out on a Veriti Thermal Cycler (Thermo Fisher). PCR products underwent electrophoresis and visualization on SYBR safe-stained 1% agarose gel to verify the absence of non-specific bands and ensure that amplicon sizes were as expected (Figure 3).

### Library Preparation and Sequencing

Library preparation was performed by enzymatic fragmentation of PCRs and double indexing using the NGSgo kit (GenDx, Utrecht, Netherlands) according to the manufacturer's instructions. The indexed libraries were pooled, denaturized, and diluted to a final concentration of 4 nM. The pooled library was sequenced on the MiSeq system (Illumina, San Diego, CA, USA).

### Data Analysis for KIR Gene Content Determination and HLA Genotyping From NGS Data

Indexed sequences were de-multiplexed and analyzed individually. KIR reads were mapped to the human genome reference sequence hg19 (GRCh37), and the binary alignment map (BAM) files containing the mapped reads were visualized using CLC Genomics Workbench 11 (Qiagen, Hilden, Germany).

To determine KIR gene content in NGS samples, 2 short (10–30 bp) gene-specific virtual sequences for Amplicon A and 2 for Amplicon B for all KIR genes were carefully chosen from IPD-KIR, resulting in 4 gene-specific sequences (virtual probes) for each KIR gene. These virtual probes were used to find exact match binding sites in reads from FASTQ files of NGS samples (using the CLC Genomics Workbench). This “*in-silico* hybridization” technique enabled determination of presence/absence and relative quantification of individual KIR genes (15). The virtual probes designed can recognize almost all alleles described to date. The only exception was 2 virtual probes from amplicon A that did not detect two uncommon alleles (KIR2DL1^*^020 and KIR2DS3^*^005) (16). All sequences of the virtual probes were aligned with BLAST against the human reference genome (hg19) to confirm KIR specificity (17).

The number of matching virtual probes per gene in each experiment was normalized against the total number of probes matched per sample. The results (KIR content) corresponding to the samples analyzed by NGS were compared with the results obtained by PCR-SSO. Using these data, an NGS threshold of < 1% of probes detected was established to consider that a gene was absent. It should be noted that the probe range varied considerably, but negative results were usually below 0.1% (Table 1).

**Table 1:** Average number (maximum-minimum) of gene-specific virtual probes and normalized data on raw data in samples with presence and absence of specific KIR genes using the 300-cycle MiSeq Reagent Kit.

	**Average number of virtual probes detected**		**N**^****°****^ **probes per gene / total N**^****°****^ **of probes detected** *100	
	**POSITIVE**	**NEGATIVE**	**POSITIVE**	**NEGATIVE**
2DL1	237 (621–52) *n =* 184	3.5(3–2) *n =* 2	5.8 (11.5–2.0) *n =* 184	0.096 (0.146–0.0469) *n =* 2
2DL2	190 (702–20) *n =* 101	3 (23–0) *n =* 85	4.6 (16.9–1.1) *n =* 101	0.074 (0.6–0) *n =* 85
2DL3	296 (942–43) *n =* 164	5 (11–0) *n =* 22	7.3 (13.0–2.2) *n =* 164	0.106 (0.29–0) *n =* 22
2DL5	761 (2253–132) *n =* 108	1 (8–0) *n =* 78	16.8 (30.8–3.9) *n =* 108	0.04 (0.2–0) *n =* 78
2DS1	151 (391–37) *n =* 78	2 (6–0) *n =* 108	3.4 (6.6–1.2) *n =* 78	0.0385 (0.17–0) *n =* 108
2DS2	199 (601–43) *n =* 101	5 (25–0) *n =* 85	4.9 (12.9–2.0) *n =* 101	0.1225 (0.75–0) *n =* 85
2DS3	158 (374–41) *n =* 68	2 (14–0) *n =* 118	3.8 (14–1.6) *n =* 68	0.055 (0.27–0) *n =* 118
2DS4	213 (583–36) *n =* 117	4 (5–2) *n =* 9	5.5 (10.6–1.6) *n =* 177	0.1 (0.207–0.05) *n =* 9
2DS5	164 (381–29) *n =* 64	6 (21–0) *n =* 122	4 (12.5 – 1.5) *n =* 64	0.1755 (0.61–0) *n =* 122
3DL1	372 (1039–54) *n =* 176	3 (5–0) *n =* 10	9.4 (18.1–3.1) *n =* 176	0.0585 (0.126–0) *n =* 10
3DS1	194 (487–48) *n =* 75	2 (7–0) *n =* 111	4.6 (19.3–1.7) *n =* 75	0.033 (0.228–0) *n =* 111
2DP1	630 (1947–107) *n =* 184	4 (7–1) *n =* 2	15 (25.5–8.0) *n =* 184	0.104 (0.16–0.048) *n =* 2
3DP1	436 (1030–92) *n =* 186	*n =* 0	10.3 (18.7–2.6) *n =* 186	*n =* 0
3DL2	324 (711–83) *n =* 186	*n =* 0	8.2 (16.7–3.7) *n =* 186	*n =* 0
3DL3	271 (684–52) *n =* 186	*n =* 0	6.6 (12.9–2.3) *n =* 186	*n =* 0
2DL4	356 (1113–61) *n =* 186	*n =* 0	9 (60.4–2.3) *n =* 186	*n =* 0

The HLA class I genotype was determined with NGSengine 2.9.1 (GenDx) using the IMGT 3.31.0 reference database, according to the manufacturer's recommendations and with all parameters set to default values. Omixon HLA Explore software (Omixon, Budapest, Hungary) was used in some samples to confirm the HLA genotype.

### Data Availability

The primers, virtual probes sequences and the PCR conditions are not publicly available because are currently protected by intellectual property rights of the authors, in prevision of their potential future commercial exploitation.

## Results

### Method Design

The alignment of all KIR genes was downloaded from the IDP-KIR database to enable design of PCR primers sufficiently generic to simultaneously amplify the total of KIR genes. This database was also used to design the specific virtual probes. In addition, specific primers had to be designed for genes showing little or no amplification with the generic primers. Sequence analysis corroborated amplification of all KIR genes using the specific sequences. The KIR primers were then combined with the primers for PCR amplification of the classic class I HLA genes, and the relative percentages were adjusted to obtain an appropriate number of reads for proper genotyping of both gene systems. Lastly, the cutoffs used in the validation procedure were established by analysis of 50 samples.

### Technical Validation of the Multiplex LR-PCR for Class I HLA and KIR Genotyping

Fifteen samples did not meet the minimum requirements for analysis and were discarded as technical failures during library preparation, regardless of KIR or HLA genotype (6 using the 300-cycle kit and 9 using the 500-cycle kit). Overall, the genotype was obtained in 186 samples with the 300-cycle MiSeq Reagent Kit v2 and in 147 samples with the 500-cycle MiSeq Reagent Kit v2. The validation study involved 4 runs, 2 with the 300-cycle kit and 2 with the 500-cycle kit. The study using the 300-cycle kit (2 × 150 bp sequence reads) yielded a cluster density of 926 ± 12 and 650 ± 16 K/mm^2^ with a passing filter of 91.62 ± 1.24 and 92.86 ± 1.95, respectively, in 2 independent sequencing runs of 96 indexed libraries. The study using the 500-cycle kit (2 × 250-bp sequence reads) gave a cluster density of 1,059 ± 18 and 731 ± 17 K/mm,^2^ with a passing filter of 90.44 ± 0.86 and 92.85 ± 1.10, respectively, in 2 independent sequencing runs of 78 indexed libraries. When using the 300-cycle MiSeq kit, approximately 130,000 reads were needed to obtain clear genotyping results for all genes; hence, a maximum of around 250 samples can be simultaneously typed in a single MiSeq run. On the other hand, using the 500-cycle kit, approximately 110,000 reads were needed for the analysis, which means that 300 samples can be genotyped in a single run.

### Determination of KIR Gene Content Using Specific Virtual Probes

**Figure 4:**
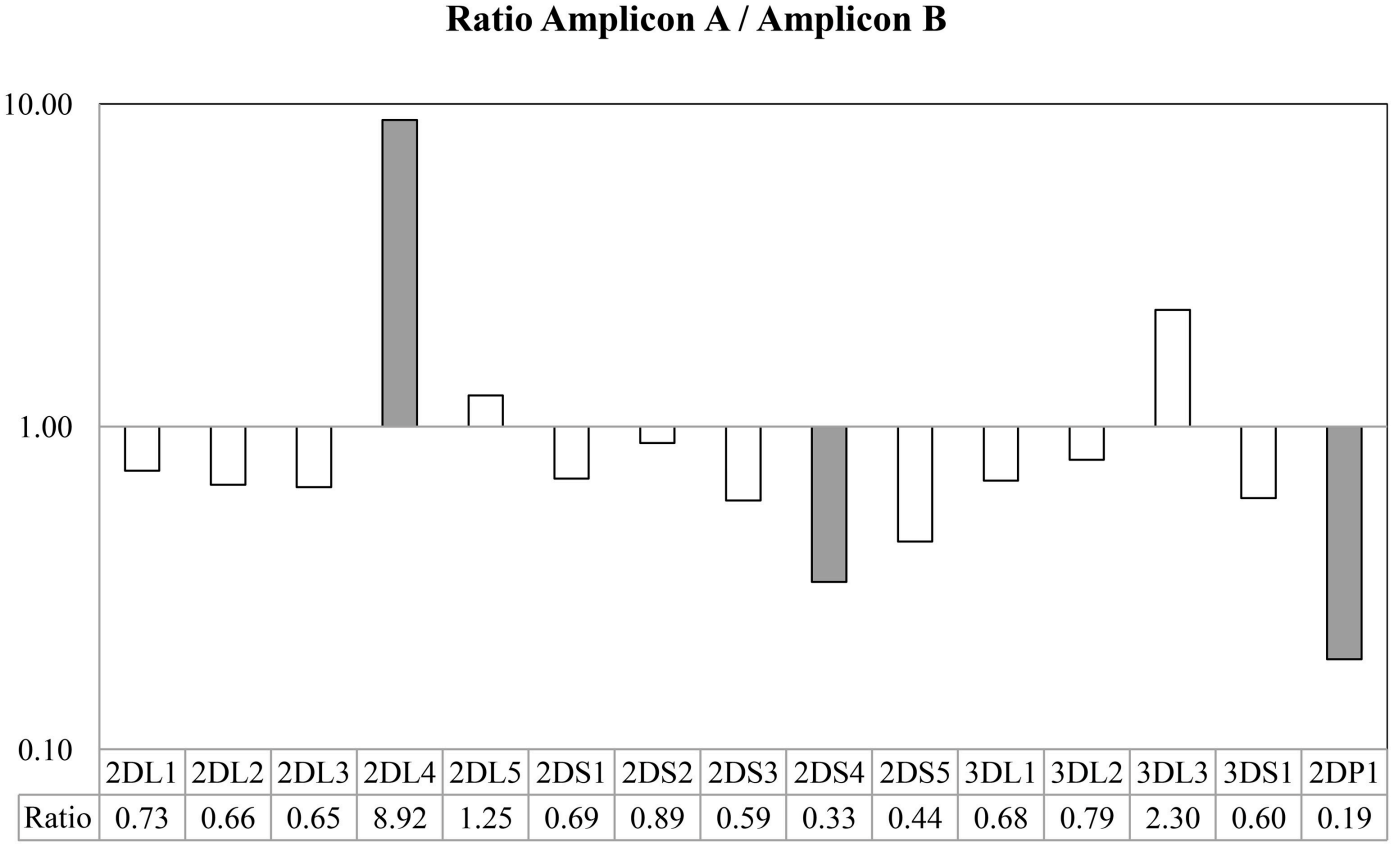
Ratio between the number of probes found in Amplicon A and Amplicon B. Taking into account the use of generic primers able to amplify all KIR genes, all amplicon loci were sufficiently balanced between them, with the exception of *KIR2DL4*, in which the Amplicon A was more extensively represented, and *KIR2DS4* and *KIR2DP1*, in which Amplicon B was more extensively represented in the multiplex.

The results based on identifying the presence or absence of all KIR genes, using gene-specific virtual probe analysis with the CLC Genomics Workbench and the established cutoffs, showed strong concordance (97.84%) with the PCR-SSO analysis, providing an identical genotype in 182 of the 186 samples studied. The following 4 discordant results occurred: In 3 samples, PCR-SSO results were positive for *KIR2DS3*, whereas *KIR2DS3* was not detected by NGS, and in the remaining discordant sample, the PCR-SSO indicated an absence of *KIR3DL1*, whereas 246 hybridizations with *KIR3DL1*-specific virtual probes detected this gene by NGS (Table 2). All 4 discordant samples were further analyzed by PCR-SSP, a second well-established and technically validated technique. PCR-SSP results were in complete agreement with the NGS findings, thereby providing evidence that NGS-based KIR genotyping was 100% accurate for all 186 samples. An average of 4,199 virtual probes matched per sample using the 300-cycle kit and 5,984 using the 500-cycle kit, after limiting the number of reads per sample to 50% to speed up the analysis without affecting the outcome. Moreover, possible overexpression of amplicon A compared to amplicon B was calculated by analyzing the number of virtual probes that belonged to each amplicon. Amplicon expression was sufficiently balanced in all KIR genes with the exception of *KIR2DP1, KIR2DS4*, and *KIR2DL4*, in which one of the amplicons was increased compared to the other, due to generic amplification of all KIR genes simultaneously (Figure 4).

**Table 2:** Results obtained in the 4 discordant samples by PCR-SSO, PCR-SSP, NGS, and the number of specific virtual probes obtained, considering 1 as positive and 0 as negative.

	**SSO**	**SSP**	**NGS**	**Probes NGS**		**SSO**	**SSP**	**NGS**	**Probes NGS**
**ID:1**									
2DL1	1	1	1	142	2DS4	1	1	1	144
2DL2	1	1	1	200	2DS5	1	1	1	210
2DL3	1	1	1	276	2DP1	1	1	1	414
2DL4	1	1	1	268	3DL1	1	1	1	184
2DL5	1	1	1	488	3DL2	1	1	1	294
2DS1	1	1	1	146	3DL3	1	1	1	180
2DS2	1	1	1	170	3DS1	1	1	1	214
2DS3	1	0	0	6	3DP1	1	1	1	322
**ID:2**									
2DL1	1	1	1	173	2DS4	0	0	0	11
2DL2	1	1	1	241	2DS5	1	1	1	327
2DL3	1	1	1	268	2DP1	1	1	1	529
2DL4	1	1	1	362	3DL1	0	0	0	0
2DL5	1	1	1	966	3DL2	1	1	1	407
2DS1	1	1	1	409	3DL3	1	1	1	269
2DS2	1	1	1	201	3DS1	1	1	1	426
2DS3	1	0	0	8	3DP1	1	1	1	349
**ID:3**									
2DL1	1	1	1	287	2DS4	0	0	0	7
2DL2	0	0	0	0	2DS5	1	1	1	367
2DL3	1	1	1	354	2DP1	1	1	1	690
2DL4	1	1	1	395	3DL1	0	0	0	4
2DL5	1	1	1	944	3DL2	1	1	1	364
2DS1	1	1	1	334	3DL3	1	1	1	251
2DS2	0	0	0	4	3DS1	1	1	1	436
2DS3	1	0	0	8	3DP1	1	1	1	478
**ID:4**									
2DL1	1	1	1	172	2DS4	1	1	1	150
2DL2	1	1	1	254	2DS5	1	1	1	236
2DL3	1	1	1	255	2DP1	1	1	1	578
2DL4	1	1	1	596	3DL1	0	1	1	246
2DL5	1	1	1	796	3DL2	1	1	1	428
2DS1	1	1	1	220	3DL3	1	1	1	298
2DS2	1	1	1	262	3DS1	1	1	1	224
2DS3	0	0	0	2	3DP1	1	1	1	480

*Discordant results highlighted in dark gray PCR-SSO, polymerase chain reaction sequence-specific oligonucleotide; PCR-SSP, polymerase chain reaction sequence-specific primers; NGS, next generation sequencing*.

### HLA Class I Genotyping

In the paired-end 2 × 150 sequencing approach, average coverage for HLA across all samples was 378 reads and mappability was 76.02%. Genotype concordance was 99.46%, with only a single difference: HLA-B^*^07:05:01:01 performed by SBT was detected as HLA-B^*^07:06:01 by NGS. The sample was re-analyzed using Omixon HLA Explore to rule out a software problem and the same result as that found by NGSEngine software was obtained. Study of the typing discordance showed that the mismatch between B^*^07:05:01:01 and B^*^07:06:01 is located in exon 5, a region that is not covered by SBT. Therefore, the NGS result was taken as correct, and 100% concordance was established.

In the 2 × 250 approach, average coverage for HLA across all samples was 345 reads, mappability was 62.77%, and the same genotypes were obtained (Table 3).

**Table 3:** Number of reads, mappability, and read depth obtained from HLA class I sequencing using the 300-cycle or 500-cycle MiSeq Reagent Kit.

**Genes**	**N^**°**^ of reads**	**Mappability %**	**Read depth**
**300-Cycle MiSeq REAGENT KIT**			
HLA-A	8676.45	70.79	373.51
HLA-B	8894.60	79.13	401.31
HLA-C	7569.11	78.14	359.22
MEAN	8380.05	76.02	378.01
**500-Cycle MiSeq REAGENT KIT**			
HLA-A	9941.22	58.34	347.96
HLA-B	10408.61	66.50	396.98
HLA-C	7475.52	63.47	290.315
MEAN	9275.115	62.77	345.08

## Discussion

Over the last few years, impressive progress has been made in NGS techniques. This new high-throughput technology has begun to show its value in clinical practice, as more and more laboratories are successfully applying it in HLA typing. Nevertheless, incorporation of NGS-based KIR genotyping into routine practice has been delayed. This task is still performed by PCR-SSO or PCR-SSP, mainly because there is no available commercial kit or user-friendly analysis software for this purpose. In an attempt to fill this gap, we developed and validated a protocol based on NGS technology for genotyping the 14 KIR genes, 2 KIR pseudogenes, and all the classic HLA class I genes simultaneously.

Validation of the method was performed with 186 samples previously typed by PCR-SSO, and concordance was initially 97.84%. Nonetheless, analysis of the discordant samples with another validated technique led to the conclusion that the NGS results were correct in 100% of the samples. Of note, the 3 false-positive *KIR2DS3* detected by Luminex technology had adjusted values in the gray area (mean = 0.362, with a cut-off of 0.288). There was also a false-negative result for *KIR3DL1* with PCR-SSO technology. This error may have resulted from the presence of an unknown KIR allele with a mismatch at the binding site of the PCR-SSO specific *KIR3DL1* probe (18).

A large number of samples was included in the present validation compared with other validations of NGS KIR genotyping methods (19, 20), and it was performed with both 300- and 500-cycle sequencing, which proved to be equally useful in terms of accuracy. This finding has cost implications, as the 300-cycle MiSeq Reagent Kit is much less expensive than the 500-cycle kit.

The analytical design presented has several advantages: (1) The multiplex PCR enables amplification of all the KIR and classic HLA class I genes in one tube, (2) Amplification of full-length HLA genes enables high-resolution genotyping without ambiguities, (3) With amplification of full-length KIR genes, allelic resolution can be obtained when adequate software becomes available, and (4) Use of 4 probes for KIR analysis increases confidence in the results. Of note, PCR-SSO usually has only 1 probe for each microsphere (equivalent to detection of 1 KIR gene).

The difference in the number of probes found per sample according to presence or absence of a gene was significant enough to establish appropriate cutoffs. However, in a few samples, the number of probes found when a gene was negative was higher than expected due to the huge number of reads involved in the analysis. To avoid errors due to variations in the read numbers per sample, we performed the calculations using percentages; that is, we quantified the percentage of probes belonging to each gene to eliminate variability due to differing read numbers per sample. Using this normalized approach, determination of the cutoffs to further establish the method was more reliable.

To date, 2 groups have developed NGS-based KIR genotyping approaches—Norman et al. (19), using exome capture and Maniangou et al. (20), using LR-PCR and subsequent sequencing—thus highlighting the increasing interest in characterizing KIR gene sequences. In addition to NGS-based KIR genotyping techniques, both the above-mentioned groups have developed software for high-resolution KIR genotyping. However, these require further refinement, as some discrepancies were detected when the two high-resolution approaches were used to obtain the genotype in the same 30 DNA samples (20). In contrast to these strategies, the method presented here is the first that enables simultaneous genotyping of all genes in the KIR cluster and concomitant genotyping of the ligand—HLA class I—using NGS technology, an accurate approach that is less costly and with higher throughput capacity than these previous methods. In our laboratory, the implementation of this technique has meant a saving of approximately 50% in reagents' cost and hands-on-time procedure, compared to the separately HLA class I typing by NGS and KIR by PCR-SSO.

Implementation of our multiplex NGS method for large-scale KIR plus HLA class I genotyping will provide a reliable tool with several potential applications; for example, selection of the most appropriate donor for allogeneic HSCT in cases of hematopoietic cord blood or partially-matched related donor (haploidentical) transplantation. The main demand for KIR genotyping comes from fertility and reproductive health centers, always in combination with HLA-C genotyping. In addition, reports are emerging of an association between KIR gene content and several conditions, such as infectious diseases, cancer, and reproductive failure (21–25).

Our future work in this line will focus on analysis of the samples reported here with our own KIR-specific software (currently in development) to reach allelic resolution. However, high-resolution KIR genotyping may require changes in some aspects of the method presented, such as new calculations to determine how many samples can be simultaneously genotyped in one MiSeq run. Adjusting the balance between HLA class I and KIR primers may also be needed, as conclusive results may require higher coverage of the KIR amplicons.

In conclusion, 100% concordance was obtained in the technical validation of the KIR genotyping method presented, based on specific virtual probes. The added advantage of simultaneous high-resolution HLA class I genotyping makes this technique an attractive candidate for further development as an effective replacement for the commonly used methods for KIR genotyping, PCR-SSO and PCR-SSP.

## Author Contributions

LC performed the technique design and development, performed KIR and HLA genotyping by next-generation sequencing, performed the interpretation and analysis of data, and wrote the manuscript. FV designed the study, supervised the technique development, and commented on the manuscript. MH commented on the manuscript. JC designed the study, supervised the technique development, and commented on the manuscript. All the authors have approved the manuscript for publication.

### Conflict of Interest Statement

The authors declare that the research was conducted in the absence of any commercial or financial relationships that could be construed as a potential conflict of interest.
